# Cultural experiences in the framework of “cultural cities”: measuring the socioeconomic impact of culture in urban performance

**DOI:** 10.1186/s40410-022-00189-8

**Published:** 2022-12-31

**Authors:** Pau Rausell-Köster, Sendy Ghirardi, Jordi Sanjuán, Francesco Molinari, Borja Abril

**Affiliations:** 1 Research Unit in Economics of Culture and Tourism, Department of Applied Economics, University of València, Av. Tarongers S/N, 46022 Valencia, Spain; 2 Department of Methodology of the Behavioural Sciences, University of Valencia, Av. Blasco Ibañez, 21, 46010 Valencia, Spain

**Keywords:** Cultural experience, Cultural impact, Urban development

## Abstract

This article defines "cultural experience" and places it in a holistic conceptual model; “the cultural city” where it plays a relevant role in improving the performing of cities. The conceptual model combines the basic elements of the heritage city, the smart city and the creative city. The city is interpreted from a threefold perspective; as a repository of resources, as a connective interface, and as the setting for citizens' life and social and professional experiences. In this context, each of these perspectives incorporates culture in a different way, enabling different models of value creation and different processes of production and reproduction of this value. In each of the urban models described above, production processes that combine symbolic, physical, financial, social, human and cultural capital in different ways and urban strategies are implemented to provide cultural experiences that ignite transformative effects through several spillovers. That means that culture, in its different dimensions, regains the role of a raw material and becomes the point of origin to activate development processes and improve urban performance. The integration of the dimensions of the heritage city, the creative city and the smart city through an enabling context is the core proposal of the “cultural city”. In alignment with the New European Agenda for Culture, we deepen the analysis in the specific spillovers on wellbeing and quality of life, citizen engagement and urban renewal as the backbone of a set of external effects of cultural experiences. In the final part of this article, we test the plausibility of this speculative proposal through some empirical evidence. We develop an OLS model with proxy indicators, that could be considered transitional indicators, for the three different potential strategies (heritage, smart, creative). The findings support the assertion that it is conceivable that the supply of cultural experiences through a variety of tactics (heritage city, smart city and creative city) can account in part for the growth of European cities in the years after the 2008 financial crisis. These strategies have contributed to the good performance of the urban device in a way that is positive, not negligible (accounting for around 50% of the variance in productivity) and statistically significant. The provision of a context that increases the cultural experiences for citizens has clearly improved the performance of European cities, and we develop some conceptual and empirical mechanisms to explain and measure the socioeconomic impacts of these processes.

## Introduction

There is no doubt that the city, as a device for human interaction and a mechanism for generating wealth, has been remarkably successful. The key to the city's success and persistence lies in the fact that it satisfies human needs with high efficiency levels, and when it does not, mechanisms appear to generate the necessary changes to transform itself. As Jane Jacobs stated more than 50 years ago, "Cities contain the seeds of their own regeneration” (Jacobs 1986). The very economies of agglomeration make inefficiencies and insufficiencies easy for citizens to be expressed and for policymakers or market agents to visualise and receive. The city appears as a "formula" with undoubted success in a long historical perspective (Sorribes 2012), and with a good forecast, as shown by the historical evolution of the urbanisation rate and the estimation that in the mid-twenty-first century nearly 70% of the world's population will live in cities.

As the Shanghai Manual (United Nations, 2012) makes clear,^1^ people gravitate towards cities not only for economic opportunities, but also looking for better education and an uninterrupted flow of ideas, information, and culture. Marxist literature claims that the city rescues people from the “idiocy of rural life” *(Manifesto of the Communist Party*), and it was also in the industrial cities that the dream of a new social order was forged and reinforced. The city has always been the neural centre of freedom, culture, and political and institutional innovation in its broadest sense. The exchange of ideas and experiences, the cultural "mix" that is consubstantial to cities, has meant an enormous positive externality for society as a whole, to the point of Jane Jacobs' affirmation "The city, the wealth of nations", which perfectly summarises this powerful idea.

In this article, we are going to test whether “cultural experiences” have effects on individuals and communities, influencing their perceptions of the city itself and, more importantly, their values, their feelings about their own identity and belonging, their behaviours, and their relationships with others, as well as the effect of these changes in urban performance. Our initial intuition is that urban cultural engineering, defined as the technique for the production of cultural experiences in the urban context, which manipulates symbolic (arts and culture, senses and meanings), material (cultural infrastructures) and technological contexts, could become a very powerful tool for social transformation, influencing the general model of urban performance, including its economic framework.

## Cultural experience at the centre of the analysis

However, if we are interested in delving into the impacts of culture beyond its economic classification, we will have to look at the impact generation process, considering the concept in all its complexity. What we seek with this approach is an operational definition of the basic process that activates and generates these processes of transformation and change in order to try to articulate plausible sequences of causality of these impacts. In his early work, Matarasso (1997) spoke of the social impacts of participation in the arts in a broad sense. He did not use the term ‘participation’ as an euphemism for community arts, but he interpreted broadly and failed to provide a precise definition. According to the UNESCO (2012), cultural participation can be defined as “participation in any activity that, for individuals, represents a way of increasing their own cultural and informational capacity and capital, which helps define their identity, and/or allows for personal expression”. Such activities may take many forms—both active, such as creating art or even volunteering for a cultural organisation, and passive, such as watching a movie—and may occur through a variety of formal or informal channels, including the internet. The notion of the prosumer—term coined by Alvin Toffler in the 80's to describe the increasing integration of consumers into the process of cultural production (Hesmondhalgh 2010)—and the lesser univocity between generator and consumer of culture recommend that the analysis should not focus on the concept of cultural participation, but on that of cultural experience. Access to cultural content loses its traditional passive, appreciative character and becomes a form of creative appropriation by the user (Valtysson 2010).

A “cultural experience” can be defined as the generation, emission or reception of information flows with symbolic content, usually expressed through artistic grammars, that have the explicit and more or less deliberate intention of having some kind of *resonance* on our cognitive, emotional or aesthetic dimension or our perception of our location in a social body. A cultural experience is a concrete act of cognitive, sensory and emotional appropriation of the world around us, the intensity and quality of which depends on material, psychological and social issues, as well as on our own cognitive and cultural capital.

In this context, we are applying the concept of *resonance* from the German philosopher and sociologist H. Rosa, who states (Bialakowsky 2018) that resonance is the opposite of alienation and has four crucial characteristics; (a) one is in resonance with something when one feels affected by it (b) the subject reacts to it—the psychological concept of self-efficacy, (c) the experience has a transformative capacity on individuals of greater or lesser intensity or of greater or lesser duration in temporal terms, and (d) resonance is not controllable and cannot be approached in a purely instrumental way; it is elusive, meaning that you cannot anticipate that it will actually happen even if you fully control the context, and its obtainability cannot be taken for granted (Susen 2020)*.* According to Rosa, resonance can be defined as “a form of world-relation, in which subject and world meet and transform each other”. The emergence of resonance is possible only ‘through af ← fection and e → motion [sic], intrinsic interest and expectation of self-efficacy’, entailing the construction of a meaningful, dynamic, and transformative rapport between actors and their environment (Susen 2020). It is important to note here that the transformative effects of resonance are beyond the control of the subject: when something really touches us, we can never know or predict in advance what we will become as a result of this.

### The cultural experience in an integral vision of the city

The non-disposability and moment-like character of resonance does not mean that it is completely random and contingent. There are structured ways to generate resonances through artistic action and participation, and ultimately cultural projects and policies are production functions of cultural experiences. However, these resonances have a very unstable chemistry, as their transformative effects do not follow a stable causal logic over time and space. Urban cultural policies constitute a more or less coherent approach based on instrumental rationality, and the city as human engine is a contextual space where the probabilities of concrete projects becoming real cultural experiences, with their associated resonances, are multiplied.

A recent UNESCO document underlines that the spatial, economic and social benefits of culture on the city are achieved through six sets of transition variables (UNESCO and World Bank 2021) that act as enabling tools for impacts. The six categories of creative city enablers that have proven critical to translating culture into spatial, economic and social benefits are: urban infrastructure and liveability, contexts to improve the skills and produce innovation, social and financial networks and technical support, inclusive institutions and friendly regulations, some sense of uniqueness through attractive storytelling and a digital environment. Further analysis of these transition indicators could provide us with better social control and improve the effectiveness and efficiency of cultural policies and projects.

With the same intention, we have taken another explanatory route. The city can take benefit from the processes of ignition of cultural experiences through three basic mechanisms (Sorribes 2012): (1) the city as a repository of heritage elements accumulated and superimposed throughout history, or as a way to generate and broadcast stories that generate resonance (2) the city as an engine for the exchange of ideas, which multiplies the possibilities of interactions that require cultural experiences (Pareja-Eastaway 2020), and (3) the city as the vital scenario where most people carry out their personal, family and professional activities and are exposed to cultural experiences (Mellander et al. 2012).

**Fig. 1:**
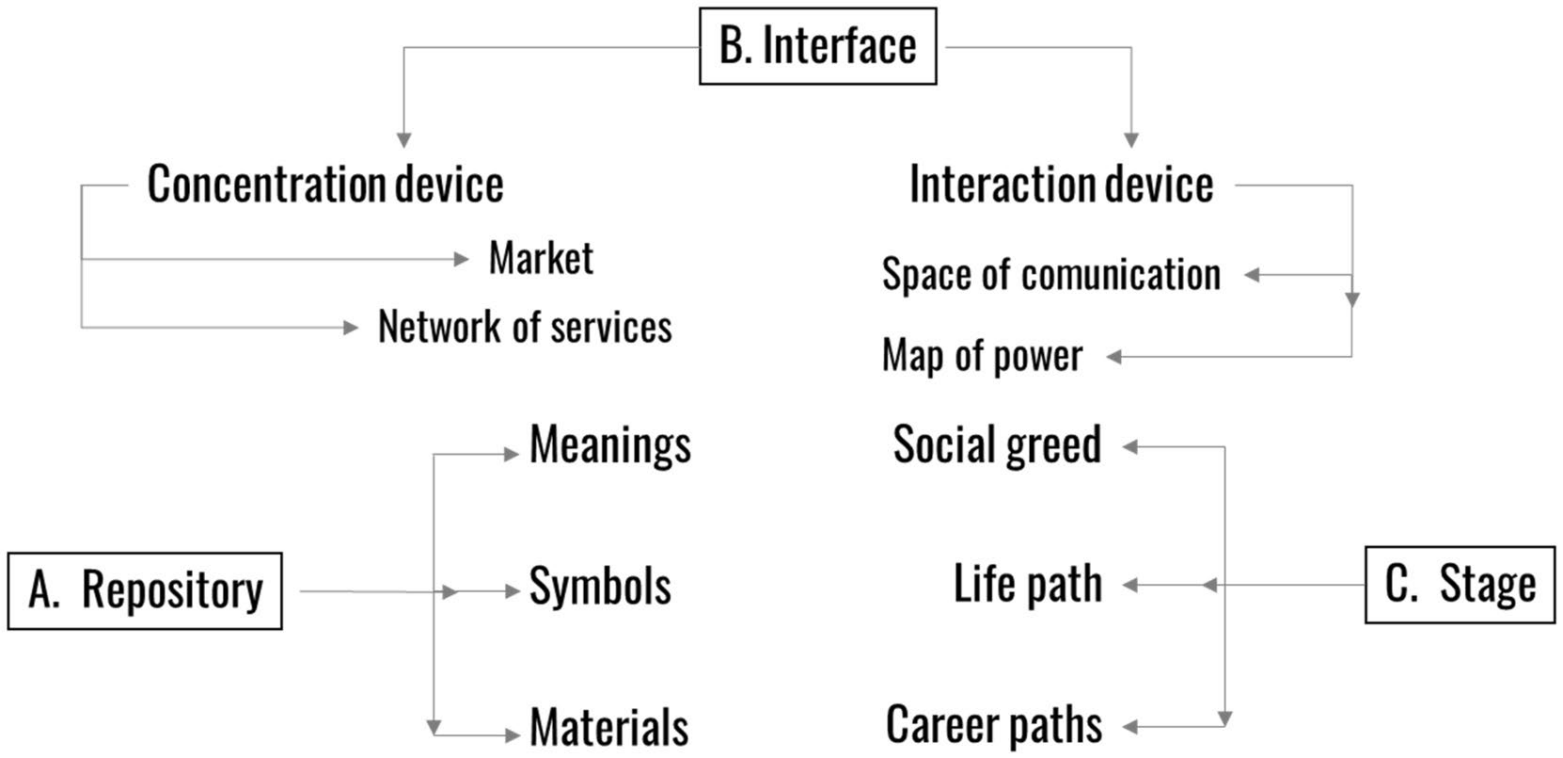
Conceptual Map. An integral view of the relations between culture and the city. Source: Own elaboration

These dimensions of the city are intertwined and articulate its social, political, symbolic, and economic fabric, as shown in the conceptual map below. According to our hypothesis, the activation of cultural experiences through any of these mechanisms will generate social, cultural and economic value, consequently improving the efficiency of the city as a "social artefact" to a greater or lesser extent. In the final part of this article, we make an instrumental simplification and assume that the greater efficiency of the "urban engine" can be approximated through an indicator such as the variation in productivity. The view we defend is that since the 2008 crisis, European cities have used some of these strategies, with varying degrees of instrumental rationality and intuition, to improve urban efficiency (Fig. 1).

## The three faces of the cultural city

The "cultural city" as a space and support for the cultural experiences of individuals becomes a relevant variable to explain the success of cities. There is extensive literature from different disciplinary fields on the location of culture and creativity in urban complexes. From the late 1980s until the onset of the crisis, different theories successfully pointed, specifically and in a renewed way, to the cultural dimension of cities (Zukin 1995) as an opportunity element to be addressed by local development strategies (Evans 2001; Florida 2002; Landry and Bianchini 1995).

Culture—understood as the set of cultural experiences that are activated in a given territory over a period of time—is interlinked and generates value in different ways, which are described in the following paragraphs.

### The city as a repository of resources: The Heritage City

The first perspective from which the concept of a city can be approached is as a geographical space where a large number of resources are concentrated (Scott 2001). This large storehouse of resources can be used to fulfil various functions. There is no doubt that one of the most important factors in the success of some cities is the dense accumulation of resources, a stock of accumulated wealth and historical capital gains deposited over time and materialised in urban assets. From the perspective of the accumulation of cultural resources, heritage cities are urban spaces that have managed to identify and recognise the value of material and symbolic resources from the cultural field and that, through a regulatory and normative process, maintain certain levels of protection and conservation. It is a type of urban organisation in which economy and culture have fused together, in a way that economic outputs are subject to ever-increasing injections of aesthetic and semiotic meaning, while the culture that is consumed is produced more and more by profit-seeking firms in the commodity form (Scott 2014).

It should be noted that urban assets, although they refer especially to the material dimension of the city, are not limited to the physical artefacts that make up cities, such as the grid of streets, buildings, gardens, monuments or public and private facilities. These should be added to the value of the iconic elements and the stories or meanings associated with the material elements. In this sense, the city can be seen as a container for the meanings attached to its material contents where the capacity to generate value is often much more related to the discourses than to the physical elements. In post-industrial cities (Scott 2014), value is increasingly generated through discourses, narratives, and information flows, rather than through the production of material goods. Therefore, cultural experiences happen when the physical elements of the city interact with its symbolic heritage elements and their meanings.

The narratives of an urban space constitute more than a brand, as they contain a set of physical and socio-psychological attributes and beliefs that can be considered as inputs to social, cultural, and economic processes. These resources have the same or even greater capacity than material resources to generate collective value and shape the *sense of place*. Moreover, such discourses are a constituent part of the cultural and cognitive capital of the people who inhabit, use or visit the sites and consequently condition their behaviours and ways of relating to each other and to the space (Table 1).

**Table 1:** The city as a repository of resources. Heritage city. Source: Own elaboration

Dimension: Repository of resources	Primary mode of value creation	Modes of production/ reproduction	Urban concept
Materials	Uses of heritage for economic, social or cultural value creation (mainly—but not exclusively tourism)	Investment in heritage creation or restoration, access and tourist infrastructures, signalling, labelisation	Heritage City
Iconic	Urban brand and marketing strategies Incorporation of symbolic value into value creation processes	Generation of emblematic elements and condensers of meaning. Slogans Production of icons	
Meanings	New meanings by comparison, similarity, contrast, hybridisation	Storytelling through films, literature, media…. Investment in cultural events, festivals	

The heritage city enhances the ability to attract or develop new and higher-order functions, increase internal efficiency (Camagni et al. 2015) and achieve economies of scale through the resignification of its material attributes. In order to achieve the resignification through new narratives, the construction of new heritage, the valorisation of existing heritage or the creation and/or revitalisation of icons to improve the average productivity of the city, the rate of return on invested capital must exceed the average urban productivity. This is possible through the reuse or heritage resources for higher value-added activities, including but not limited to tourism.

### The city as an interface for exchange and communication: The Smart City

The second dimension in which we place the processes of value generation is the concept of the city as an interface that enables the concentration of resources and interaction. The concentration of resources in a limited geographical space is the necessary condition for the activation of certain processes, without the concurrence of which the success of an urban space would not be possible (Concilio et al. 2019). While the concentration of producers, workforce and consumers in a physical or virtual space is necessary for the articulation of a market, it also poses logistical, economic and social challenges related to organisation, regulation and service provision, without which it would collapse (Florida et al. 2017). In other words, the concentration of material resources forces the search for technological, organisational, social, economic or spatial solutions to overcome its propensity to collapse. Productivity improvements in this dimension are achieved through the concept of the Smart City.

The Smart City can be understood as a set of innovation processes that improve urban life in terms of living conditions, economy, mobility and governance primarily—although not necessarily—through information and communication technologies (ICT) (Anthopoulos and Reddick 2016). The Smart City response has been the use of technological innovations and data analytics applied to the city as a connective interface, driving away congestion costs and improving the efficiency of processes and the effectiveness of urban service delivery.

In the realm of interaction spaces, the city articulates both spaces of conflict (competition), where competing interests and alternative use of resources and patterns of appropriation of public and private spaces are settled, and spaces of communication (collaboration). Density is both an agitator of conflict and a fertiliser of communication. “*We find ourselves immersed in an epoch of problematic transition, in which culture and the city are alternatively defined as spaces of conflict or spaces of hope”* (Segovia and Hervé 2022). The first of these two approaches defines the political arena of the city and shapes certain power relations that are channelled into a concrete institutional architecture and shape a concrete symbolic representation (Concilio et al. 2019). The material shaping of the city itself is a more or less subtle representation of power relations and hierarchies (political, religious, economic and cultural), with its town halls, churches and banks in the centres (Monnet and Jérôme 2011).

One of the key elements in this context is that the city enables the concentration of human capital, which as we know from the Romer-Lucas models (Romer 1986) is the central element of economic growth theories. To explain why cities attract human capital, three theories can be identified (Storper and Scott 2009): (a) Florida's "creative class" theory, (b) research by Glaeser and others that identifies a broad set of amenities—educational or cultural—and weather conditions, and (c) Clark's notion of the city as an entertainment machine that offers parks, museums, art galleries, orchestras and landmark buildings. However, dynamic cities are also great attractors of people because of their ability to offer well-paid jobs, as they have higher levels of productivity derived from agglomeration economies. Therefore, the smart city locates cultural experience in the dynamics of agglomeration and the mechanics of density, in the exchange of ideas, in people-to-people communication and interaction, and in the generation of opportunities for connections that would otherwise have been improbable. The smart city as a facilitator of the generation of cultural experiences is based on its ability to take advantage of the concentration of niche demands and cross-fertilisation and serendipity (Table 2).

**Table 2:** City as connective interface

Dimension: Connective interface	Primary mode of value creation	Modes of production/reproduction	Urban concept
Concentration mechanism	Economies of scale and agglomeration	Investment in digital infrastructures, mobility and commuting upgrades Development of monitoring and control services	Smart City
Mechanism of interaction Spaces of conflict Communication spaces	Competitions of ideas Economics of diversity Cross-fertilisation Serendipity	Political arena and power relations. Designing governance models Scene of ideas Development of mechanism of interaction	

Agglomeration economies are the result of both economies of scale and the network economies that develop when firms and people are located close to each other. They are therefore related to spatial proximity and, Glaeser, (2011) states, can be formulated as a reduction of transport costs in a broad sense, i.e. transport costs related to goods, but also to people and ideas. Today, cities have a productivity advantage for different reasons related to the circulation of ideas and people rather than costs, in contrast to the industrial cites of the nineteenth century. In this sense, digitalisation, urban mobility and commuting speed become relevant elements to approach the efficiency of physical and virtual interaction processes.

### The city as a stage for the life trajectories of individuals and communities: The Creative City

The third dimension to which we wish to refer is the concept of the city as the setting for the vital, personal, professional and social trajectories of the people who inhabit it. With urbanisation levels expected to reach 70% by 2050 (United Nations 2019) the city is becoming the setting where most of the planet's inhabitants' life events take place and, consequently, the main determinant of our individual levels of wellbeing, utility and/or happiness. Although economic factors have a strong impact on subjective wellbeing in low-income territories, there are evolving cultural changes in territories with higher levels of development, with people attaching greater importance to self-expression and freedom of choice (Inglehart and Welzel 2005). Other authors suggest that pleasure, engagement and meaning are the three main components of life satisfaction (Peterson et al. 2005). These factors are closely linked to the satisfaction of individuals' cultural rights.

The ability of cities to satisfy the symbolic needs of their residents defines their success, which is rediscovering its original meaning once more. As a result, this capacity is becoming more and more dependent and more connected to the cultural ecosystem. The city as a space for creation and experimentation generates value by activating sufficient stimuli to enable people’s integral development through the exercise of creativity, the pursuit of pleasure and the enjoyment of rich and multiple experiences. The key lies not so much in the functionality and efficiency of the economic device as in the potential of the social fabric and the space for the development of personal and social relations—in short, in the liveability of the urban environment (McArthur and Robin 2019). The richness and density of this network is conditioned by its capacity to stimulate a sense of identity, commitment to the community and belonging, and promote participation and trust in others (Table 3).

**Table 3:** The city as a living and working environment

Dimension: Stage for life trajectories	Primary mode of value creation	Modes of production/ reproduction	Urban concept
Space for individual creation and experimentation	Human capital externalities Innovation	Generation of public and private spaces for creative and sensorial experiences Artistic and digital literacy Facilitation of innovation processes Professional opportunities	Creative city
Space for the development of personal and social relations	Impacts of the sense of identity, commitment to the community, sense of belonging, participation Meaning, pleasure	Place making Production of values (sustainability awareness, inclusion, gender gaps…) Urban facilities and public goods	
Space for the development of professional relations	Attractiveness Productivity	Generating an enabling environment for the creation of enriching work opportunities High wages High ROI	

If we want to maximise the utility of our life trajectories, we are no longer guided by purely instrumental rationality, but also by the expressive values of exchange and mutual benefit. This is what creates the tension between the physical or constructed city (*la ville*) and the lived city (*la cité*) (Sennett 2018). The ethics and values linked to the increasing centrality of the human condition in the urban setting extend spatially, socially and economically and enable the emergence of new activities, some of which have economic value but also drive technological innovation and community development. Sustainable development, creativity, transparency, participation, accountability, technology, and engagement are the pillars of new social activities and new productive sectors (Rausell-Köster et al. 2012). Citizens who are aware, well informed and in control of their freedoms wish to develop their professional and life trajectories through activities such as social innovation, creative activities, proximity economy, collaborative economy, circular economy, care activities, green economy and the economy of the common good because they allow them to find a sense of commitment, pleasure and meaning in their daily actions. The determinants of behaviour in the new emerging activities respond to a new hierarchy of values associated with cultural practices: pleasure, the desire for innovation, relational (versus transactional) consumption and free exchange, critical thinking, personal development, solidarity, cooperation, networking, the value of diversity and beauty, the sense of justice, participation and the importance of the recreational and vital dimension beyond purely economic benefit (Boix-Domènech and Rausell-Köster 2018).

The urban concept that captures this vision is the Creative City as formulated by Landry and Bianchini (1995), who tried to identify what could improve people's lived experience of cities. Today, we know that the concentration of cultural and creative activities in a given territory changes the logic and functioning of its economic dynamics in a deeper and more complex way than we had previously assumed and affects the potential range of personal experiences available to citizens in a determining way.

We also know that the centrality of creativity and innovation is changing the role of economic organisations and human resource management models, and we know that a liquid labour market is taking shape around this fact, combining liberating trends for human work that enable enriching personal development experiences with realities that tend towards extreme precariousness and self-exploitation. The Creative City refers to the attractiveness and competitiveness of the urban environment based on cognitive and symbolic elements whose main mechanism for generating added value is turning creativity into market, aesthetic or social innovation. Scott introduced the notion of "cognitive-cultural capitalism" (Scott 2014), to argue that we are entering a period marked by a distinctive third wave of urbanisation based on cognitive skills and cultural assets. The economic value of urban activities is subject to increasing injections of aesthetic and semiotic meaning, while the culture that is consumed is increasingly produced by for-profit companies in the form of commodities (Scott 2014). Professional opportunities in the creative sector become a good indicator to identify the Creative City.

Combining Jacobs' ideas about cities with Schumpeter's ideas about innovation, it is argued that innovation and risk appetite do not only take place in cities, but require cities to occur. (Florida et al. 2017). However, one of the potential pitfalls is that innovation and equity are not two spontaneously cooperating issues (Pileri 2015).

The risks of the Creative City are identified in the possible slide towards the *society of the spectacle*, the trivialisation of the symbolic dimension or the growing pressures associated with the commodification of all cultural experiences, including those that fulfil an important social function. Recent critiques also refer to phenomena of social polarisation that are seen to be caused by the occupation of certain urban spaces by the creative class such as social segmentation in cities, gentrification, segregation and the exclusion of middle-class families from urban centres—*the new urban crisis—*(Florida 2017).

But with all its possible distortions and problems, the creative city is the desired setting for a population that is increasingly educated and demanding in all its expressive, social and professional experiences.

### The conceptual model of the Cultural City

Cultural experience is associated with several types of positive effects, ranging from achieving innovation and lifelong learning objectives to fostering social cohesion and health and wellbeing (Sacco et al. 2018). The New European Agenda for Culture, whose strategic objective is to harness "the power of culture and cultural diversity for social cohesion and wellbeing", focuses on a structural model based on the dimensions of health and wellbeing, urban and territorial renovation and people’s engagement and participation (European Commission 2018), which are also addressed by the MESOC project^2^ (2021). MESOC adapts and further develops a method of “transition based” impact assessment derived from a previous UNESCO Chair publication, building a structural model of the Societal Dimension of Culture, as defined by one of the strategic objectives of the European Agenda.

Through cultural experience in facilitative contexts, individuals learn and reconfigure the codes that underlie cultural meaning. Cultural experiences bring about changes in individuals (Soren 2009), impacting on knowledge, skills, attitudes, values, emotions, beliefs, relationships, and states of mind. From the perspective of cultural experiences, participation in cultural experiences within a community generates impacts that ensure wellbeing and progress in the era of post-industrial economy, in areas that go beyond traditional spillover (Sacco et al. 2013).

**Fig. 2:**
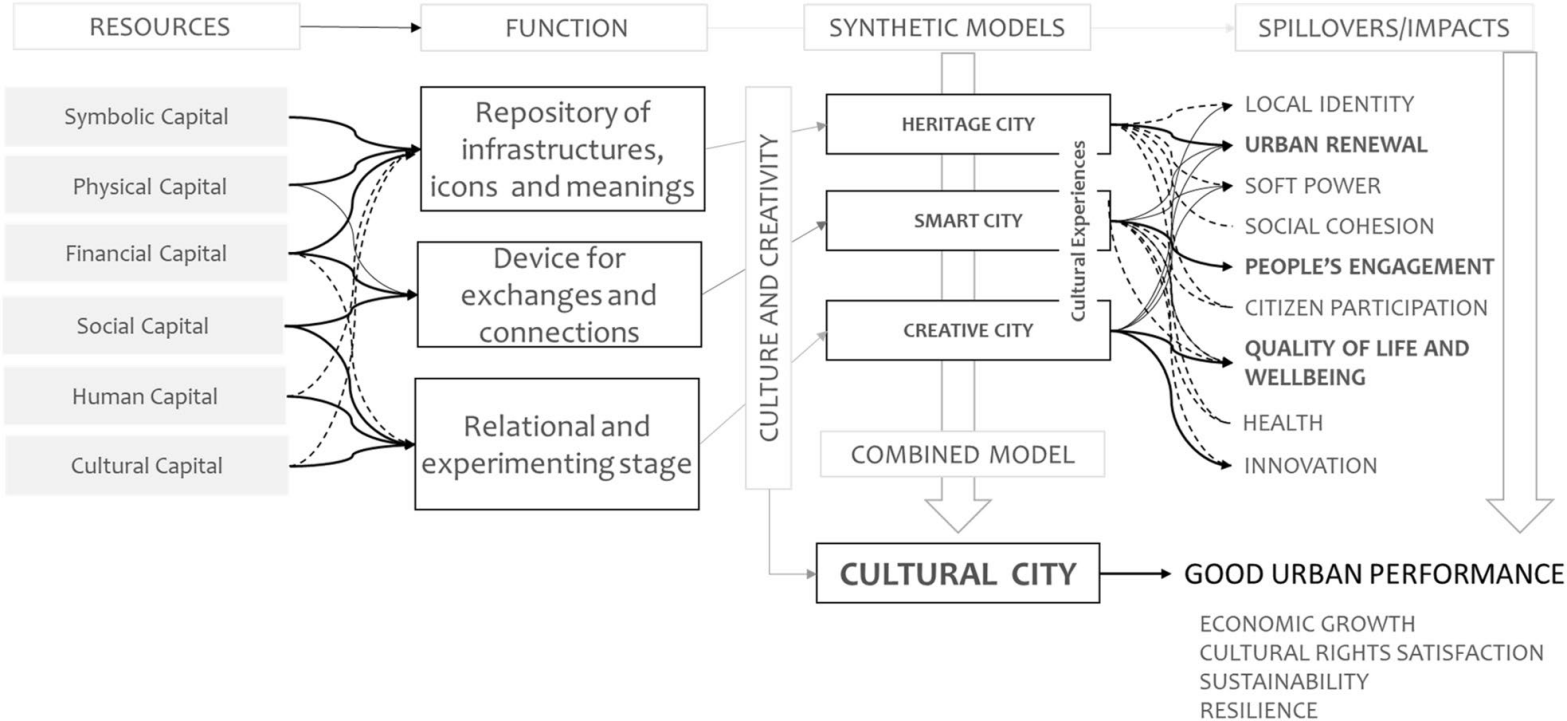
The “Cultural City” model

Each of these paradigms shows, through a certain dynamic perspective, the relationship between culture and the city. In each of the urban models described above, production processes in which symbolic, physical, financial, social, human, and cultural capital is combined in different ways and urban strategies are implemented to provide cultural experiences that ignite transformative effects through several spillovers. That means that culture, in its different dimensions, regains the role of raw material and becomes the starting point for the activation of development processes and the improvement of urban performance. The integration of the dimensions of the Heritage City, the Creative City, and the Smart City in an enabling context is the core proposal of the Cultural City (Fig. 2).

The New European Agenda for Culture (European Commission 2018), whose strategic objective to harness “the power of culture and cultural diversity for social cohesion and wellbeing", focuses on an impact generation model that directly connect an individual or collective experience with arts and culture to three main societal impact domains: health and wellbeing, urban renovation and social cohesion.

#### Quality of life, health and wellbeing

It is acknowledged that culture influences people’s behaviour, their self-esteem and, ultimately, their health and wellbeing. Aspects related to health and wellbeing can be directly connected to the concept of the city as the setting in which our life trajectories develop, with the cultural and creative dimension being the ingredient that facilitates or hinders a "good life". Our perceptions of health and wellbeing are directly influenced by our lifestyle, how stimulating and creative our work is, the quality and density of our social and family relationships, the intensity of our cultural practices and the meaning of our actions. All these aspects are related to culture and creativity. In this sense, the perceptions around health and wellbeing become indicators of whether or not that "good life" materialises.

The 67th World Health Report of the World Health Organization synthesizes the findings of over 3500 studies on the role of the arts in the prevention of illness, the promotion of health and the management and treatment of illness across people’s lifespan (Fancourt and Finn 2019). The report highlights how the components of the cultural experience, i.e. aesthetic engagement, involvement of the imagination, sensory activation, evocation of emotion, cognitive stimulation, social interaction, and physical activity, can trigger psychological, physiological, social, and behavioural responses that are themselves causally linked with health and wellbeing outcomes.

Certain studies note that cultural participation is the second most important determinant of a person’s psychological wellbeing, preceded only by the absence of disease, with a significantly stronger impact than variables such as income, place of residence, age, gender or occupation (Grossi et al. 2012). Moreover, the studies reveal that the impact of culture on subjective wellbeing is far more relevant in contexts of high cultural supply and cultural engagement than in circumstances of low endowment and low participation (Tavano Blessi et al. 2016). As a result, two factors appear to be critical in terms of culture as an urban planning tool for individual and collective wellbeing: cultural vibrancy in terms of policy initiatives, use of facilities and activities, and an individual and social propensity to experience cultural activities and goods.

In 2010, cultural participation and cultural heritage density and policies became part of the Measures of Equitable and Sustainable Wellbeing Index by the Italian National Institute of Statistics, which attempt to go beyond GDP (Cicerchia 2018). Over the last few decades, many governments disillusioned with the traditional use of GDP or income as a measure of their citizens’ welfare have started focusing on wellbeing. Governments from all over the world have been introducing new indices of progress in which the concept of culture appears as a wellbeing determinant to guide their policymaking (Hall et al. 2010).

Cultural-led development strategies can therefore be defined as sets of actions operating on a broad variety of urban cultural assets (from the cultural heritage to the visual arts, from museums to theaters, etc.), whose final objective is the maximization of residents’ well-being (Perucca 2019).

#### Urban and territorial renovation

The interplay between urban and territorial renovation, culture and cultural initiatives and urban governance modes (Degen and García 2012) is widely recognised as a developmental key for cities to offer a high quality of life at both the spatial and social levels (Evans 2005). Everything started in Europe in the mid-1980s, when post-industrial cities sought to revive former industrial, contaminated and waterfront sites and their city centres as they aimed to establish themselves in the new arena of the global market and cities started looking at cultural planning and programming as strategies to enable economic development and promote spatial and social regeneration.

Urban renewal, although not exclusively, is about the perception of the city as a repository of physical and symbolic elements. The impact of culture is the capacity to regenerate and re-signify spaces with culture and creativity, either by developing new cultural functions on existing spaces or by improving the functionalities and uses of culturally significant spaces. As stated in the 2018 Davos Declaration on high-quality Baukultur for Europe, “we urgently need a new, adaptive approach to shaping our built environment; one that is rooted in culture, actively builds social cohesion, ensures environmental sustainability, and contributes to the health and wellbeing of all” (European Ministers of Culture 2018). The Urban Agenda Partnership for Culture and Cultural Heritage, created in November 2018 under the Urban Agenda of the EU, has the objective of defining actions to improve regulation, financial capacity and data/knowledge exchange of EU urban authorities that share the common goal of improving the management of their historical built environment and preserving the quality of urban landscapes and cultural heritage. An Orientation Paper (Partnership on Cultural and Cultural Heritage 2019) was published in November 2019 and the revised Leipzig Charter, which was published more than 20 years after the signature of the original one to promote the adoption of integrated urban development policies and set out the key principles behind them for the first time in a single EU document, reaffirm the notion that culture is at the core of any sustainable urban development, including the preservation and development of the built and non-built cultural heritage. Cities have used “built culture” for urban regeneration through reactive models focused on providing a response to the decline of the industrial city or on the possibility of making better use of the opportunities available, trying to attract global tourism, investment or fluxes of creative citizens in the framework of the redefinition of their position in the global hierarchy or in circumstantial and adaptive planning (Boix et al. 2017).

#### People’s engagement and participation

It goes without saying that a city with high levels of citizen participation and engagement in both political and cultural life is a city with a good performance. The absence of engagement and participation might be interpreted as a lack of freedom of choice, which jeopardizes the pursuit of positive freedom (Sen 1999). It is now recognised that cultural experience has an impact on empowerment, providing people with the social tools they need to comprehend the behaviours and motivations of others, as well as the confidence they need to act socially. There is ample evidence of the impact of cultural experiences on citizen engagement and participation and, more generally, on social cohesion. Studies focused on the impacts of participation in the field of culture have been carried out by renowned authors like Matarasso (1997), Stanley (2006) and Brown and Novak-Leonard (2013), among others. Indeed, there have even been examples where culture has been used as a political tool for conflict resolution and the activation of pro-social behaviour (Cala Buendía 2010). The indivisibility between life and work, the way in which new technologies are altering our ways of communicating and relating, or the tensions derived from the local and global demands that converge in the city are some of the bridges between people’s engagement and the city as a stage (Segovia et al. 2015).

However, when discussing cultural participation and urban policies, it is important to address not only impacts but also the strategies for accessing culture. Ever since the introduction of contemporary cultural policies, participation in culture has been a primary goal (Tomka 2013). For example, the theme of participatory governance applied to cultural heritage is a topic of great interest in the European context (Sani 2015).The issue of access to culture and social inclusion has been analysed by scholars like Laaksonen (2005) who stressed the importance of adopting a cultural rights approach. Brown et al. (2011) studied the modalities of participation, identifying five main typologies according to the degree of involvement. In the last few years, we have been witnessed a “participative turn” that is changing the panorama and dynamics of cultural policy (Bonet and Négrier 2018). Therefore, the implications of people’s participation for governmental cultural policies is becoming relevant in the current debate (Jancovich and Bianchini 2013). With the new societal trend of "prosumerism", an increasing number of people feel that they have the right to have their voice heard and they exercise that right to the best of their ability under their specific circumstances. This paradigm, which shifts from passive to active, is affecting different aspects of society and appears reflected in each of the three urban models presented in this paper.

## Some evidence of the plausibility of the Cultural City model

In the following paragraphs we will try to provide, without further empirical pretensions, the plausibility that the model of the cultural city as a combined proposal for the functionality of culture in the creative, heritage and smart city can be a useful explanatory model. The logic is as follows; if it can be empirically proven that we can explain the performance of a set of cities from combinations of different creative, heritage and smart city strategies, then we have some clues that the concept of “cultural city” is comprehensive and complete enough to explain the dynamics of cities from the perspective of culture.

### Methods

In order to try to give plausibility to the analytical proposal developed in the previous paragraphs, we make a gross simplification. Our hypothesis is that between 2008 and 2018, European cities improved their performance by using, either deliberately or intuitively, some combination of strategies that use culture, and more specifically “cultural experiences”, as a central element in the value generation processes in one of the models described; that is, through the Heritage City, the Smart City or the Creative City.

The second step in this simplification is approaching "improved urban performance" through the proxy of a variation in labour productivity.

The third step is approximating the use of each of these strategies through very simple synthetic indicators. The Heritage City strategy is approximated with the indicator of the number of museum visitors. The Smart City strategies are approximated with the variables of the number of ICT graduates—digitalisation—and the agility in commuting—interaction. Finally, the Creative City strategy is proxied by new jobs in the creative sectors (career opportunities) and risk-proneness (proxy for innovation).

However, the purpose is not to elaborate a complete econometric model capable of fully explaining the economic growth of cities, but to determine whether these elements have a significant impact and, if so, whether it is a positive one. This is a first empirical approach to confirm or disprove our hypothesis. We are aware that there are already numerous studies in the academic literature on productivity growth that are extremely accurate and that incorporate variables such as the capital stock, the rate of capital depreciation, the rate of growth of technology, etc. These variables, however, are hardly obtainable in a reliable way at the local level. While all these additional variables, among others, would be necessary for a rigorous sophisticated model that attempts to explain productivity growth accurately and robustly, this is not the purpose of this article. We would like to stress that the added value of this work does not lie in the robustness of its empirical evidence but in the consistency and plausibility of its theoretical proposal.

#### Model

The core idea of the model is to test whether the three components of the cities we have conceptualised (Heritage City, Creative City, and Smart City) contribute to their economic growth and development and, if so, to what extent. As a proxy for the concept of economic growth, we will use the cumulative change in productivity between 2008 and 2018. An ordinary least squares regression (OLS) is applied, with the following equation:

1 $${\Delta Productivity}_{i}=\,{\beta }_{0}+ {\beta }_{1}\cdot {heritage}_{i}+{\beta }_{2}\cdot {creative}_{i}+{\beta }_{3}\cdot {smart}_{i}+{\varepsilon }_{i}$$

where, for a given city *i*, the estimated increase in productivity depends on the sum of its indicators in the areas of heritage, creativity and smartness as explanatory variables, multiplied by their coefficients ($\beta$), and added to the intercept (${\beta }_{0}$) and a random error (${\varepsilon }_{i}$) that responds to variables not observed in the model.

The indicators defining the heritage, creative, and smart components are explained in the following section.

#### Data

Obtaining indicators and comparable data at the local level is always a major challenge, as they are not always accessible and sometimes, they do not even exist. To address this problem, the database has been built using a combination of different sources. However, a limitation of this method is that the city coverage of the different indicator panels does not always coincide. The Cultural and Creative Cities Monitor (Montalto et al. 2019), the European Digital City Index (Bannerjee et al. 2016) and the OECD all provide different indicators. The first covers a sample of 190 cities; the second, 60; and the third, another 60. The intersection of the samples from these three sources results in 50 cities from 23 European countries, which make up the sample for this analysis.

Regarding the dependent variable, productivity at the local level is taken from the OECD, which defines it as GDP per worker in USD at constant prices and constant purchasing power parity (PPP). Based on these data, the indicator used in the model corresponds to the cumulative change, in percentage points, between 2008 and 2018.

There are three explanatory variables, which we have called *Heritage*, *Creative* and *Smart*.

- *Heritage* is composed of a single indicator: museum visits per 1,000 inhabitants, which is obtained from the Cultural and Creative Cities Monitor. The original source is Eurostat (Urban Audit) and the data refers to the period 2011–2017.
- *Creative* is composed of two indicators:
  - New jobs in creative sectors per 100,000 inhabitants. Derived from the Cultural and Creative Cities Monitor, and originally collected from Eurostat (Regional Statistics). It includes three sub-indicators that are weighted equally: new jobs in arts, culture and entertainment enterprises; in media & communication; and in other creative sectors. The data corresponds to the period 2010–2016 and to the NUTS 3 regional level in which each city is inserted.
  - Willingness to take on risk, defined as the percentage of people who disagreed with the statement “One should not start a business if there is a risk it might fail". It is taken from the European Digital City Index, which in turn takes this indicator from the 2013 Eurobarometer. The data correspond to the NUTS 2 regional level in which each city is inserted.
- *Smart* is also composed of two other indicators:
  - Commute. This variable is also derived from the European Digital City Index. It is a score that is calculated from Numbeo data and considers the average distance and travel time from home to work. Higher values represent better scores, i.e., shorter time and shorter distance, showing a better performance of the city as an interface device.
  - Annual graduates in ICT per 100,000 inhabitants. Derived from the Cultural and Creative Cities Monitor, and originally collected from the ETER project. Data corresponds to the period 2013–2015 for tertiary education.

Given the difficulties of obtaining standardised data at the local level, these indicators have been chosen because they reasonably capture some of the defining features of the conceptualised city typologies. We select visits to museums not only because museums are the most representative repositories of the heritage stock of cities in its many different forms, but also because the number of visitors is a good indicator of the enhancement of this heritage and the involvement of citizens in it. Moreover, both the emergence of new creative jobs (which measures the capacity and opportunities to exploit creativity through the local productive structure) and the willingness and open-mindedness of the population to take risks and undertake uncertain projects, ideas and initiatives (as a prerequisite for developing creativity and innovation) are variables that allow us to quantify the capacity of cities to activate creative processes. Finally, a highly digitalised environment with widespread access to and use of technological tools (measured through ICT graduates that provide the required human capital for its development) and efficient transport infrastructures that minimise the time and distance between places and allow cities to become accessible spaces of interpersonal connection are two defining features of Smart Cities, as explained above.

All the raw indicators used to construct the explanatory variables, both those from the Cultural and Creative Cities Monitor and from the European Digital City Index, are first standardised according to population. Subsequently, they have been subjected to a winsorization process in case they contained outliers. That is, if the distribution of a variable has a kurtosis greater than 3.5 and an absolute skewness greater than 2, upper-end outliers are substituted with the next highest value and lower-end outliers with the next lowest value. This process is repeated iteratively until a distribution that meets the kurtosis and skewness requirements is obtained.

This process of winsorization is followed by a min–max normalisation process, so that all indicators fall within an interval of 0 to 1. This, in addition to allowing a direct comparison of the coefficients of the three components considered in the regression (heritage, creative and smart), is necessary to aggregate variables with different magnitudes within the same score. It also applies in the case of the new creative jobs variable. This, as mentioned above, in turn considers three different indicators: new jobs in arts, culture and entertainment enterprises; in media & communication; and in other creative sectors. In order to weight these three areas equally, so that the different dimensions do not introduce biases, the min–max score is obtained first, and then averaged. The scores from the Cultural and Creative Cities Monitor and the European Digital City Index have not been used directly but have been recalculated for the sample of cities used in our analysis. The equation used for the min–max normalisation is as follows.

2 $${z}_{i}=\frac{{x}_{i}-\mathrm{min}(x)}{\mathrm{max}\left(x\right)-\mathrm{min}(x)}$$

where ${z}_{i}$ is the normalised score for city *i*, ${x}_{i}$ is the original value for city *i*, and min(x) and max(x) are the minimum and the maximum value in the sample for variable x.

Table 4 summarises the descriptive statistics of the different variables incorporated in some way in the model, with the original winsorized data. The lower part of the table also shows the scores that were finally used in the model after the normalisation process within the interval from 0 to 1.

**Table 4:** Descriptive statistics

	Avg.	Std. Dev.	Max.	Min.	N
Δ∆Productivity_2008-2018_ (%)	7.75	11.68	54.01	− 13.27	50
Museum visits (per 1,000 inhabitants)	3170.91	1908.58	8645.83	0.05	50
Willingness to take on risk (% of pop)	51.28	11.05	71.50	25.20	50
New creative jobs (per 100,000 inhabitants):					
In arts, culture and entertainment	110.90	61.67	369.02	15.20	50
In media & communication	87.03	54.08	192.11	10.74	50
In other creative sectors	350.93	185.14	871.13	34.28	50
Graduates in ICT (per 100,000 inhabitants)	100.96	88.32	393.41	7.01	50
Commute (score)	2.78	0.71	4.79	1.52	50
Scores					
Heritage	0.37	0.22	1.00	0.00	50
Creative	0.46	0.15	0.81	0.15	50
Smart	0.31	0.16	0.88	0.02	50

## Results and discussion

The results of the OLS regression applied following Eq. (1) are shown in Table 5. All three components, as well as the intercept, are found to be statistically significant. All three also have coefficients with a positive sign, i.e., they are positively related to productivity growth. The magnitudes, however, differ. The largest effect corresponds to the Smart component, followed by the Creative and the Heritage component. Comparing the magnitudes of the coefficients, it can be noted that the Smart component score is responsible for 55% of the growth explained by the model, compared to 28% for the Creative component and 17% for the Heritage one. However, we should not underestimate the effects of heritage on issues that go beyond economic growth such as sense of belonging, community building or psychological wellbeing.

**Table 5:** Results of the OLS regression

	Estimate	Std. Err	t value	Pr (>|t|)	
(Intercept)	− 21.060	4.827	− 4.363	0.000	***
Heritage	13.326	5.399	2.468	0.017	*
Smart	43.200	7.388	5.848	0.000	***
Creative	22.430	8.145	2.754	0.008	**
R^2^					0.5338
Adjusted R^2^					0.5034

*Significant at 5%, ** significant at 1%, *** significant at 0.1%

The OLS model has an adjusted R^2^ of 0.5034. This means that the model only explains about half of the variability in productivity growth of cities. The limited explanatory power of the model must therefore be borne in mind. However, this should not come as a surprise. It is worth remembering that the aim is not to determine all the factors that contribute to productivity growth in cities from a holistic perspective, but only to test the effect of some of them, i.e. those conceptualised in this article. Naturally, the model leaves out a multitude of explanatory factors, ranging from the national and regional economic context to the productive structure, the embedded capital or the provision of key infrastructures.

**Fig. 3:**
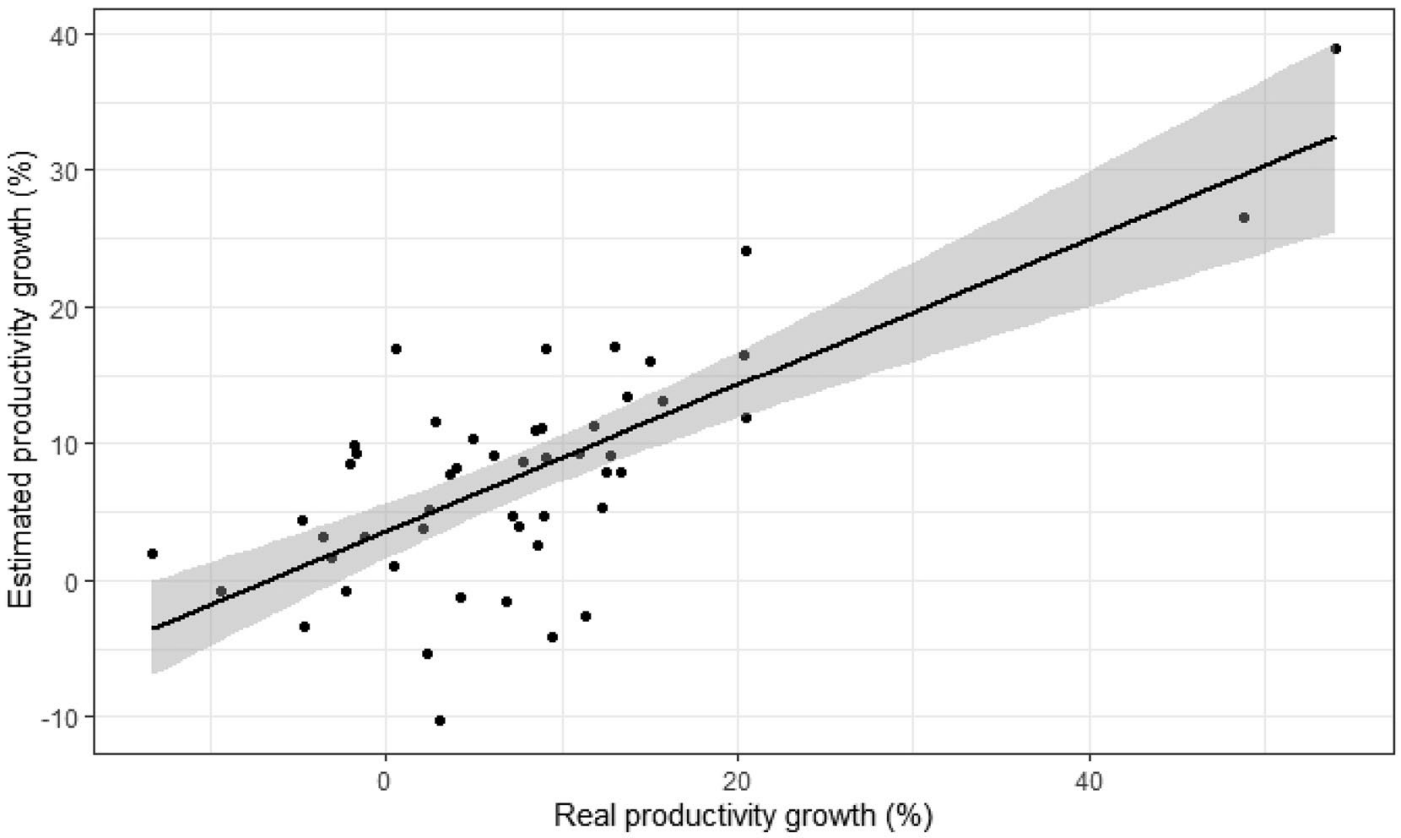
Matching model-estimated productivity growth with real growth

Nevertheless, given the enormous complexity of the phenomenon under study and the multitude of factors that are not considered, the model is still a reasonable fit. Figure 3 shows a graphical representation of the model estimation, considering the three modelled parameters, compared to the current values of local productivity growth.

The validity of the model is tested by applying a Shapiro–Wilk normality test (Shapiro and Wilk 1965). The result (W = 0.9841, p-value = 0.7322) indicates that the residuals are normally distributed, so the model is adequate. Variance inflation factors (VIF) are also checked to verify that there are no multicollinearity problems among the independent variables (Dormann et al. 2013). The values are, in fact, very low (*heritage* = 1.027, *creative* = 1.059, *smart* = 1.069), so the presence of multicollinearity is discarded.

In sum, the model provides a first empirical confirmation of our initial hypothesis. If we consider that these three components act as a driver of growth in cities, this growth may be partly due to different combinations of these components in each case. Hence, different specialisation models can be defined depending on which of the components predominates. We would therefore be talking of Heritage Cities, Creative Cities or Smart Cities.

**Fig. 4:**
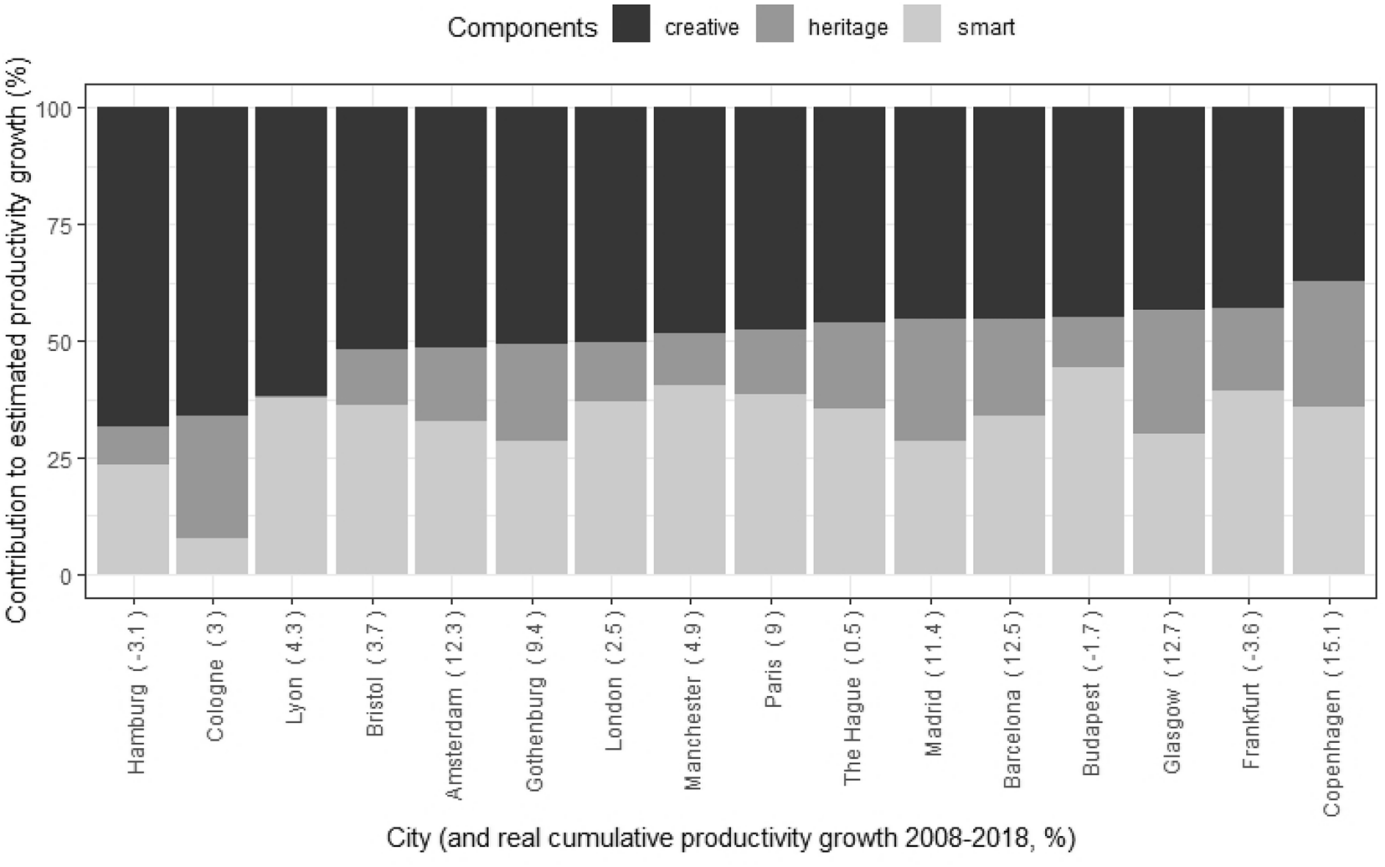
Sample cities classified as ‘Creative Cities’ ranked by share of contribution to productivity growth of creative component

**Fig. 5:**
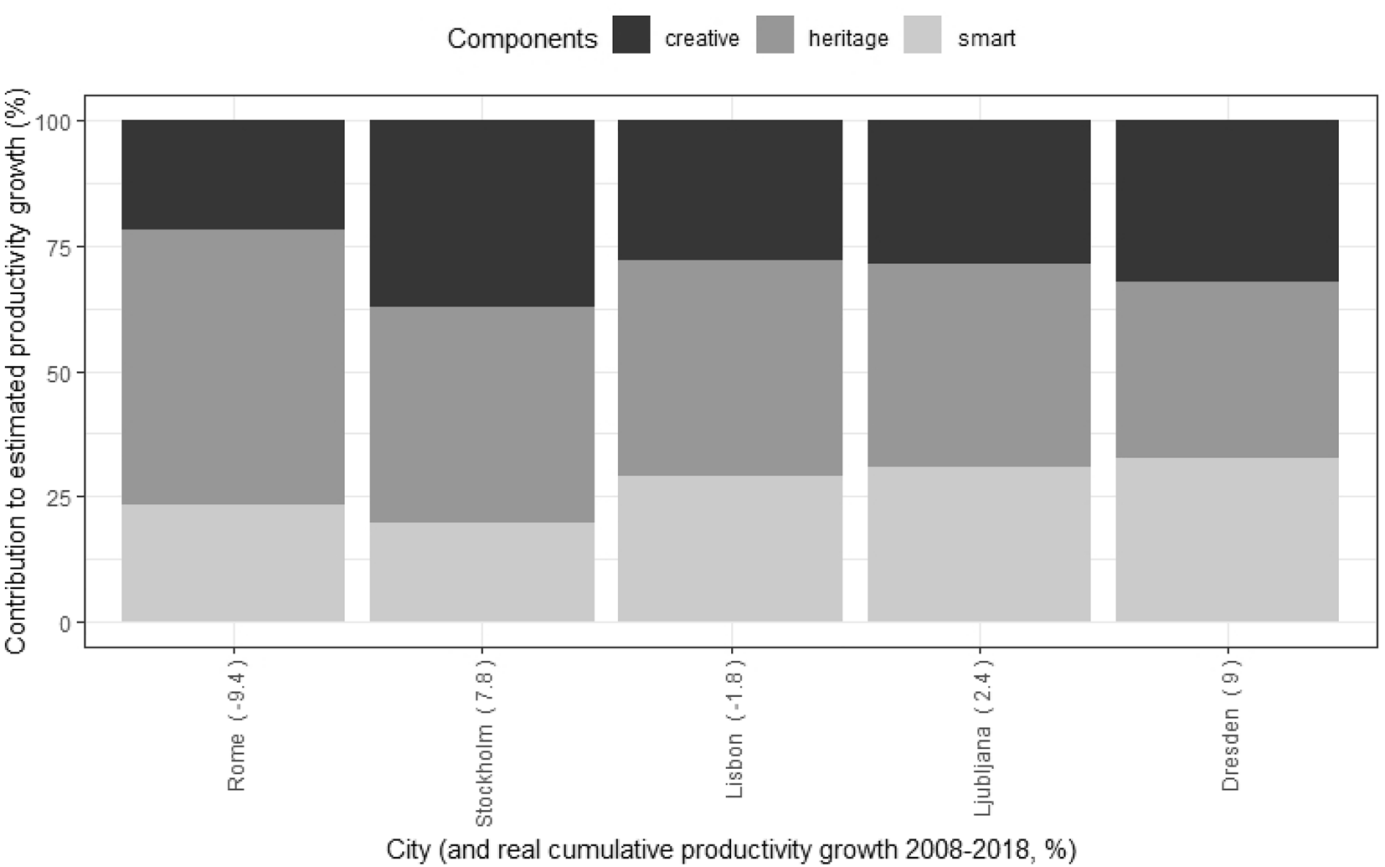
Sample cities classified as 'Heritage Cities’ ranked by share of contribution to productivity growth of heritage component

**Fig. 6:**
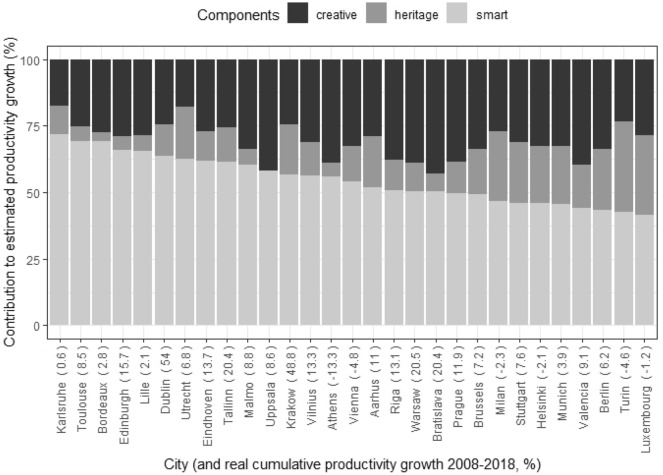
Sample cities classified as 'Smart Cities’ ranked by share of contribution to productivity growth of smart component

From our database and model estimates, we can rank the cities according to which component explains the most productivity growth in each. This results in 5 Heritage Cities and 16 Creative Cities, while the remaining 29 stand out for their Smart City component (Figs. 4, 5 and 6). The most representative city of the Heritage Cities in the sample would be Rome (55% of the Heritage component). Hamburg would be the most prototypical case of a Creative City (68% of this component) and Karlsruhe would be the most prominent Smart City (72%).

We have sufficient evidence that culture and creativity have played a relevant role in the recovery of European cities in the aftermath of the 2008 crisis. This effect has been articulated in different ways. We have been able to define a conceptual structure that includes the three main strategies *(Heritage City, Smart City, and Creative City*) and have also found an indicative way to measure their effects and test their plausibility. The conceptualisation of the “*Cultural City"*, which integrates all three approaches, opens up new avenues for research and comparison in other geographical spaces, other scales and other periods.

From the point of view of policy recommendations, increasing the provision of cultural experiences is a strategy that improves the performance of cities. The second recommendation is that the social values generated through the Heritage City can be enhanced and formulas beyond tourism should be sought, and the third is that digitalisation and the improvement of urban switching speed, both of which are quite dependent on local authorities, have a considerable impact that is likely to become even greater as a result of the Covid-19 pandemic.

## Conclusions

It seems that we can accept that the analytical approach we set out in the first part of this article is plausible. It is plausible that part of the growth of European cities in the post-2008 crisis period can be explained by the provision of cultural experiences through different strategies (Heritage City, Smart City and Creative City). These strategies have statistically and positively contributed in a significant way to the good performance of the urban device, accounting for around 50% of the variance in productivity. The interpretative framework that we have called "the Cultural City" represents a more or less balanced combination of the Heritage City, the Smart City and the Creative City. We are of course aware that we are not dealing with a complete and definitive test that validates this new framework. Rather, we are making an approximation to the plausibility of the proposal through partial and circumstantial evidence that so far fits.

In a way, we are identifying some transitional indicators that make it possible to connect cities’ cultural experiences and performance improvement processes. It must be understood that although our dependent variable is the variation in the productivity of the labour factor in the cities, this variable approximates the good performance of the cities and includes the impacts on different dimensions that beyond the strictly economic (for example, healthier citizens or those with a better perception of their wellbeing are also more productive agents in the economic sphere and more efficient in the processes of participation or collective action and reflection). Thus, variables such as risk propensity, the number of visitors to museums or the number of ICT graduates anticipate the fluidity of the transmission processes between cultural experiences and the impacts on good urban performance. These transitional indicators are not limited to those that fit statistically into the model (or are available to us) but point to a wider family of variables that enable the transformation processes that take place through cultural experiences. These transitional indicators need to be investigated more intensively, as they define the transmission mechanisms between the policies and projects that produce cultural experiences and their final impacts on the economy, culture or society.

Although the ways of generating value are very diverse, the main ways in which we should focus in future research are those that materialise through the improvement of citizens' health and wellbeing, those that are generated by a greater commitment to the community (enhancing understanding and capacity for action; creating and retaining identity; modifying values and preferences for collective choice; building social cohesion; contributing to community development and fostering civic participation), and those that materialise in the processes of urban regeneration with social impacts through “placemaking” processes and economic value generation, placing high added value activities in new refurbished urban spaces or through real state or tourism impacts. Another possible area of research improvement would be to connect individual preferences with cultural experiences by testing their effects on socio-economic impacts. This research will be further developed in the future with the AU Culture application that tries to measure individual impacts of cultural participation (see the Resources of the MESOC project).

If we look at levels, it seems that the Smart dimension is the one that has contributed the most to growth, with an impact that is twice that of the Creative dimension and almost three times that of the Heritage dimension. These differences probably have to do with the times and circumstances we are living in. Since the 2000s, cities have invested in technology to enhance their competitiveness. One of the thematic objectives of EU Cohesion Policy during the 2014–2020 period was to enhance access to, and the use and quality of information and communications technology, including developing products and services and strengthening applications. The EU eGovernment Action Plan (2016–2020) currently sets out concrete actions to accelerate the implementation of existing legislation and the related uptake of online services. The digital transition is reshaping public services, and it is clear that its impact is very significant. Nine out of ten cities report that their services have improved as a result of digitalisation (ESPON 2017). Changes in urban mobility have also taken an important leap forward in this period. The uptake of digital solutions and changes in mobility shortens the time and lowers the cost of obtaining information, contacting other people, accessing cultural experiences and carrying out administrative procedures. Two in three cities have seen an increase in the uptake of specific services, including culture, as a result of digitalisation and two in five have even reported a substantial increase (ESPON 2017). Our database includes some medium-sized cities where the Smart strategy is clearly central, such as Karlsruhe, Toulouse, Edinburgh, Bordeaux, or Lille. This strategy clearly predominates in more cities and is probably the one where the relationship with culture and creativity is more diffuse and the transformation is more systemic.

Within the scope of the Creative Cities strategy, we can identify large European capitals that are also major centres of creativity and culture such as Paris, London, Madrid, Amsterdam or Copenhagen. Finally, the Heritage strategy is more prevalent in cities with significant historical and artistic heritage such as Rome, Lisbon or Ljubljana. The reason why the Heritage City strategy has a lower impact on the model is probably because the way to capitalise the impacts is either through tourism, an activity with low average productivity, or through the increase of real estate value, an activity that has gone through a crisis during the period considered.

In conclusion, the provision of contexts that increase citizens’ cultural experiences has clearly improved the performance of European cities and this study suggests a series of conceptual and empirical mechanisms that can help explain and measure the socioeconomic impacts of these processes.

## Data Availability

The datasets used and analysed during the current study are available from the corresponding authors upon reasonable request.

## Footnotes

1 The Shanghai Manual was established in 2011, following the 2010 World Expo in Shanghai, China. The initial purpose of the Manual was to serve as a tool to support mayors and urban managers in achieving sustainable urban development in cities.

2 MESOC is a Research and Innovation Action designed to propose, test and validate an innovative and original approach to measuring the societal value and impacts of culture and cultural policies and practices, related to three crossover themes of the new European Agenda for Culture: 1) Health and Wellbeing, 2) Urban and Territorial Renovation, and 3) People’s Engagement and Participation. The global aim is to respond to the challenge posed by the H2020 Call (“To develop new perspectives and improved methodologies for capturing the wider societal value of culture, including but also beyond its economic impact”).
